# Sense of smell, anti-ribosomal p antibodies and neuropsychiatric manifestations in childhood systemic lupus erythematosus

**DOI:** 10.1186/1546-0096-12-S1-P312

**Published:** 2014-09-17

**Authors:** Fernando A Peres, Barbara Longhi, Aline Tamires Lapa, Karina de Oliveria Peliçari, Nailu Angelica Sinicato, Mariana Postal, Renata Barbosa, Roberto Marini, Simone Appenzeller

**Affiliations:** 1 Department of Medicine, Rheumatology Unit, Unicamp, Campinas, Brazil

## Introduction

Neuropsychiatric involvement in cSLE has a prevalence ranging from 20 to 95% and results in significant morbidity and mortality. Multiple autoantibodies have been reported in association with NPSLE. Anti-P seem to be highly specific for SLE and might also be a marker for SLE disease activity. In SLE population the prevalence of anti-P varies from 6% a 46% depending on ethnicity, age and clinical variables analyzed. Anti-P are able to binding neuronal cells of the limbic areas that are associated whit olfaction. Decreased olfaction was observed in patients with several central nervous system CNS diseases in which immune-mediated mechanisms have been implicated. Evidence that olfactory dysfunction is an early sign of neurologic diseases makes a patient’s sense of smell of direct relevance to the neurologist

## Objectives

To assess the olfactory functions in cSLE patients compared with age- and sex-matched healthy controls, to examine the association between the sense of smell, anti-P, disease activity, damage and CNS involvement.

## Methods

Olfactory functions were evaluated using the Sniffin’ Sticks test. All individuals were submitted to a standardized neuropsychiatric evaluation. Mood disorders were determined through Becks Depression and Becks Anxiety Inventory (BDI and BAI). Active neuropsychiatric lupus was diagnosed according to ACR guidelines. In SLE, disease activity was evaluated through SLE Disease Activity Index (SLEDAI), damage through Systemic Lupus International Collaborating Clinics/American College of Rheumatology Damage Index (SLICC-SDI). Anti-P was measured by ELISA kits. p<0.05 was considered significant.

## Results

We included 61 patients (91.8% women) mean age of SLE patients was 17.51+- 3.42 years and 61 controls matched for age and sex (p=0.701). Smell deficit correlated with duration of disease (r=-0.295; p<0.05) and anxiety (r=0.279; p<0.05). A decrease in the sense of smell was observed in cSLE patients (31.14%) and controls (27.87%) (p=0.697). Not observed anosmia in both the cSLE and control groups. Patients had an average of 31.26 +- 4.47 total points at the 3 stages of Sniffin’ Sticks test while the controls had mean of 33.28 +- 5.28 points (p<0.05). The olfactory alteration was not correlated with depression (r=0.055; p=0.678), SLICC (r=0.777; p<-0.042), SLEDAI (r=-0.118; p=0.419), CNS involvement (p=0.586) and alterations olfactory (p=0.518). Anti-ribosomal P antibodies were identified exclusively in SLE patients and were present in 15 (24.59%) of them. Anti-ribosomal P antibodies were not associated with CNS involvement (p=0.561), but when we analyzed each manifestation separately, we only observed an association between the presence of anti-ribosomal P antibodies and convulsion (p<0.05).

## Conclusion

Comparing cSLE patients and matched controls, we observed a significant decrease in the olfactory abilities in cSLE patients, which was correlated with duration of disease and CNS manifestations. Anti-P are predominantly found in the serum of patients suffering from lupus with neuropsychiatric involvement, especially psychosis in adults, therefore, monitoring longitudinal these patients is needed for see whether will present during the disease neuropsychiatric manifestations and if decline olfactory might be a useful and easy tool for the physician in early diagnosis of CNS involvement in autoimmune diseases.

## Disclosure of interest

None declared

